# E-Learning: from useful to indispensable tool

**DOI:** 10.1590/S1516-31802012000600001

**Published:** 2013-01-18

**Authors:** Alessandro Wasum Mariani, Ricardo Mingarini Terra, Paulo Manuel Pêgo-Fernandes

**Affiliations:** I MD. Postgraduate Student in the Discipline of Thoracic and Cardiovascular Surgery, Faculdade de Medicina da Universidade de São Paulo.; II MD. Attending Physician in the Thoracic Clinic, Hospital das Clínicas, Faculdade de Medicina da Universidade de São Paulo, and Instituto do Câncer do Estado de São Paulo.; III MD. Associate Professor in the Department of Cardiopulmonology, Faculdade de Medicina da Universidade de São Paulo.

E-learning, meaning electronic learning, is a teaching model based on use of technology with the fundamental characteristic of not requiring the student’s physical presence. In other words, it consists of learning through studying material in computer environments and on the internet or other electronic media, where the teacher, if any, remains at a distance.^1^

Classically, there are two types of e-learning: distance learning and computer-assisted learning. Distance learning uses information technology to take learning to different locations. Computer-assisted learning, also known as computer-based training, uses computers as the sole teaching tool, through different forms of media, without the presence of a teacher or tutor, even at a distance.^2^ These two types are becoming increasingly integrated as the internet develops.

Medicine is a field of knowledge in which there has been high investment in developing e-learning tools. This is very clear just from the observation that in the PubMed database alone, 111 studies were published on this topic in 2011 (from searching using the key words “e-learning”, “medical” and “education”).

Some recent examples include the following:

- Richardson et al.^3^ published their experience from using an online tool over a two-year period in three countries in North America, at a total of 24 different sites, with the aim of teaching about musculoskeletal radiology. They reported that even though different platforms were used (PC, Mac and Smartphone) and different internet bands were used for connections, the experience was very productive for both the students and the educators;
- A more daring initiative was developed by Joshi et al.,^4^ who evaluated a tele-education program in the state of Pernambuco, Brazil, consisting of four one-hour classes per week over a 20-month period, and covering the most essential areas of medicine, such as public health, mental health and pediatrics. After each session, the participants filled out an assessment questionnaire. There were 141 participating centers in 73 municipalities. The authors gathered a total of 3504 responses regarding the program and found that there was a high level of satisfaction, with ratings of good or excellent from 97%;
- Use of tele-education for resolving deficiencies in medical qualifications was exemplified by Agrawal et al.,^5^ who developed a distance training program for radiotherapists in order to address a shortage of such professionals in the Indian state of Uttar Pradesh. Over a two-year period, the entire residents’ program of theory classes and case discussions at the center in Lucknow, India, was shared with residents at remote locations. At the end of the two-year period, all of the educational activity and the impact on the professionals who participated in it were audited. The authors concluded that the program had been effective in training specialists at a distance.
- One very interesting Brazilian experience was Cyber Tutor, a teaching tool for dermatology that was developed for undergraduate medical teaching, using an interactive website.^6^ The topics for the classes were based on the curriculum of the Federal University of Rio Grande do Sul. Instructive clinical cases, theory classes and up-to-date bibliographic references were selected. Photographs of the lesions were obtained through selecting patients who were attended at the dermatology outpatient clinic.

This year, in partnership with the Telemedicine Sector, the Discipline of Thoracic Surgery of the University of São Paulo School of Medicine (Faculdade de Medicina da Universidade de São Paulo, FMUSP) created an online course on oncological thoracic surgery (www.cirurgiatoracica.org.br/cacto), for which the target public consisted of thoracic surgeons throughout Brazil. The course content was delivered through making available scientific articles relevant to each topic, followed by discussions monitored in a forum for approximately 15 days. After each topic had been concluded, a class recorded for the internet was made available. Another activity of this course was web conferencing to discuss cases. This was impaired, mainly because of the low average speed of the Brazilian internet.

One important component within the current context of e-learning is the repositories: institutions or associations that make available the tools, courses and programs that they or third parties produce. The following are some important examples:

- MedEdPortal, Association of American Medical Colleges (AAMC) - https://www.mededportal.org;
- The Health Education Assets Library (HEAL) - http://www.healcentral.org.

Some universities are leading similar initiatives, both for teaching their own students and for teaching extramural students. One example of this is the association between Harvard University, the Massachusetts Institute of Technology and the University of California, Berkeley, through which the website https://www.edx.org has been developed, with a variety of courses in different fields that are available worldwide.

Another important initiative is the Coursera website (https://www.coursera.org/), which currently brings together 33 universities, mostly in the United States, including some traditional names like Duke University and the University of Stanford, but also other institutions like the École Polytechnique Fédérale, in Lausanne, Switzerland, and Hong Kong University of Science and Technology. All of these institutions make courses available in different fields of knowledge, free of charge for students worldwide, while maintaining high quality levels.

The following have been indicated as some of the advantages of e-learning: ease of access and flexibility of hours; personalization of the content; the pace of learning can be defined by the student; the course content is continually available; reduction of the cost and time expended by the student, possibility of training a large number of people at the same time, possibility of covering geographically dispersed audiences, and development of self-study and self-learning capabilities among students.

Regarding the disadvantages, the following have been highlighted: occurrence of technical problems, which are still common; difficulty of some students to adapt to the digital environment; need for the student to have greater discipline and self-organization; greater delay in creating and preparing online courses, in comparison with traditional courses; the cost of implementing the structure for developing-learning programs is still high; the low quality of the internet in many places creates major limitations, particularly with regard to transmission of images and videos. Nevertheless, the greatest criticism of e-learning is the absence of direct contact and the deficiencies that this may cause, such as limitation of students’ socialization. The advocates of e-learning argue that this can be compensated through creation of virtual communities and interaction through chat systems, forums, e-mail contact, and so on.

Even with the criticisms and limitations, what is certain is that technology applied to education enables increased possibilities for knowledge dissemination. This becomes a means of democratizing knowledge and makes it possible for knowledge to be available at any time and in any place. Thus, we conclude that learning based on e-learning tools is consistent and effective, and should increasingly be present in medical teaching at all levels, from undergraduate to postgraduate. Incorporation of these tools should be regarded as desirable by everyone working in medical education.
